# Association of Ficolin-2 Serum Levels and *FCN2* Genetic Variants with Indian Visceral Leishmaniasis

**DOI:** 10.1371/journal.pone.0125940

**Published:** 2015-05-12

**Authors:** Anshuman Mishra, Justin S. Antony, Pandarisamy Sundaravadivel, Hoang Van Tong, Christian G. Meyer, Reshma D. Jalli, Thirumalaisamy P. Velavan, Kumarasamy Thangaraj

**Affiliations:** 1 Council of Scientific and Industrial Research—Centre for Cellular and Molecular Biology, Hyderabad, India; 2 Institute of Tropical Medicine, University of Tübingen, Tübingen, Germany; 3 Fondation Congolaise pour la Recherche Medicale, Brazzaville, Republic of Congo; University of Dundee, UNITED KINGDOM

## Abstract

**Background:**

Visceral leishmaniasis (VL), one of the neglected tropical diseases, is endemic in the Indian subcontinent. Ficolins are circulating serum proteins of the lectin complement system and involved in innate immunity.

**Methods:**

We have estimated ficolin-2 serum levels and analyzed the functional variants of the encoding gene *FCN2* in 218 cases of VL and in 225 controls from an endemic region of India.

**Results:**

Elevated levels of serum ficolin-2 were observed in VL cases compared to the controls (adjusted *P*<0.0001). The genetic analysis revealed that the *FCN2 *structural variant +6359 C>T (p.T236M) was associated with VL (OR=2.2, 95% CI=1.23-7.25, *P*=0.008) and with high ficolin-2 serum levels. We also found that the FCN2*AAAC haplotype occurred more frequently among healthy controls when compared to cases (OR=0.59, 95%CI=0.37-0.94, *P*=0.023).

**Conclusions:**

Our findings indicate that the *FCN2* variant +6359C>T is associated with the occurrence of VL and that ficolin-2 serum levels are elevated in *Leishmania* infections.

## Introduction

Visceral leishmaniasis (VL; Kala-Azar), a neglected tropical disease strongly associated with poverty, claims 400.000 new cases and 40.000 deaths annually [1]. VL leads to a loss of about 2 million disability adjusted life years (DALYs) every year [2]. The vector-borne infection occurs in the four distinct clinical manifestations as cutaneous leishmaniasis, muco-cutaneous leishmaniasis, VL and post-kala-azar dermal leishmaniasis [3]. VL is the severest form and severely affects visceral organs including the spleen, liver and lymph nodes [4]. Although transmission of VL has been reported in 66 countries, more than 90% of the disease burden are observed in six countries only, viz. Bangladesh, India, Nepal, Sudan, Ethiopia and Brazil [3]. Among these countries, the Indian sub-continent (India, Nepal and Bangladesh) harbours 67% of the global VL disease burden [5]. In particular, the Bihar state of India shares 50% of VL and is considered a “hot spot” of VL [6]. Inadequate vector control practice and disease management have been claimed to be responsible for the increased incidence of VL and associated mortality in India [7].

*Leishmania donovani* is the causative agent of VL in India. The organism is transmitted to mammalian hosts by infective bites of the sandfly *Phlebotomus argentipes*. *L*. *donovani* is a unicellular trypanosomatid protozoan parasite with a dimorphic life cycle between the sandfly vector (extracellular promastigotes) and the human host (intracellular amastigotes) [8]. Both developmental stages of *L*. *donovani* are coated with various secreted and membrane bound phosphoglycans. During the promastigote stage, abundant lipohosphoglycan (LPG) and gp63 are expressed, which aid immune evasion of the parasite by inhibiting the phagolysosome biogenesis in phagocytes [9]. Further, these glycoconjugates facilitate the parasite´s survival in the hostile macrophage environment [10]. However, LPG and gp36 may also serve as pathogen-associated molecular patterns (PAMPs) which are recognized by pattern recognition molecules (PRMs) of the innate system such as complement serum proteins, mannose-binding lectin (MBL), ficolins (FCN), other soluble C-type lectins and toll-like receptors [11]. Serum complement activating pattern recognition molecules act in a first-line innate defense against promastigotes inoculated by the sandfly bite. *Leishmania* parasites have developed various evasion strategies to avoid the lytic action of the complement system. The parasites use host complement proteins to escape the immune attack by entering into macrophages [12]. Mannose-binding lectin (MBL), a circulating serum protein, recognizes the carbohydrate domain of *L*. *major*, *L*. *mexicana*, and *L*. *braziliensis*. MBL binds to the surface of *Leishmania* promastigotes to opsonize the parasites. Upon binding to parasites, MBL initiates the complement cascade and provides an additional uptake mechanism of parasites by enhancing opsonophagocytosis and protects them from the immune attack [13,14] and, thus, modulates the clinical outcome of VL [15].

Ficolins are serum complement lectins that are structurally and functionally analogous to MBL [16] and, hence, expected to modify the clinical outcome of VL due to their involvement in innate immunity. Interestingly, a significant association of a distinct *FCN2* haplotype with cutaneous leishmaniasis has been reported from a Syrian population [17]. Ficolins are a group of complement activating pattern recognition molecules consisting of a collagen-like tail region and a fibrinogen-like domain (FBG) [18]. Three types of ficolins (Ficolin-1, -2, -3) of similar structure exist in humans. These types have differential tissue expression patterns and functions [19]. The role of ficolin-2, as an innate immunity component, has been studied in several infectious diseases including Hepatitis B, schistosomiasis, Chagas disease and others [16,20–22]. Ficolin-2 recognizes superficial acetylated compounds of invading pathogens by their FBG domain and initiates the lectin complement cascade [23]. The *FCN2* gene localizes to chromosome 9q34.3 (OMIM 601624) and hepatic cells predominantly express the corresponding protein. The variants in the promoter region of *FCN2* gene at positions -986A>G, -602G>A and -4A>G have been observed to modulate the circulating ficolin-2 concentration in a dose-dependent manner. The non-synonymous exon-8 variant alleles at positions +6359C>T and +6424G>T were shown to exhibit differential binding affinities to acetylated compounds when compared to the wildtype reference alleles [24]. Studies have shown that inter-individual variation of circulating ficolin-2 concentration are correlated with polymorphisms in the promoter and exon-8 regions [25].

Although it has been showed that *FCN2* gene polymorphisms and haplotypes are associated with cutaneous leishmaniasis [17], no investigations of ficolins have so far focused on VL. Moreover, we recently observed that functional *MBL2* polymorphisms and lower MBL levels confer relative protection against VL (unpublished). As ficolin-2 shares similarities both in structure and function with MBL [26], we aimed to explore the role of potentially important *FCN2* gene variants and circulating ficolin-2 levels in VL in our Indian study group. Three promoter SNPs (-986A>G, -602G>A and -4A>G) and two structural SNPs in exon 8 (+6359C>T and +6424G>T) were genotyped and studied.

## Materials and Methods

### Ethics statement

Informed written consent was obtained either from the participating individual or from the parents/guardians if an individual was less than 18 years old. The study was approved by the Institutional Ethical Committee (IEC) of the CSIR-Centre for Cellular and Molecular Biology (CCMB), Hyderabad, India. Permission was also sought for and obtained from district government hospitals.

### Study design and sample collection

This is a case-control study matched for ethnicity, sex and geographical location. All cases and controls were recruited through multiple field visits from villages located within a radius of ~120 km from the city of Muzaffarpur in the Bihar state of India. Previous epidemiological studies of VL have indicated that the Bihar state is a hot spot for VL with an average annual incidence of 2.49/1000 individuals [6]. The sample size was calculated prior to recruitment using the Open Epi platform (http://www.openepi.com/) based on the incidence rate and the risk of VL in the study area. A total of 443 unrelated subjects (218 cases and 225 healthy controls) were recruited. The mean age of VL cases was 28.7±16.7 and 35.3±16.2 in healthy controls (*P* = 0.001). No significant difference in the male/female ratio was observed in cases (125:95) and controls (122:93). The cases were determined based on the clinical features of VL in medical records issued by government hospitals in the study region. Typical clinical features of the cases included fever with rigors and chills and significant splenomegaly. Cases were tested with the rk39 leishmanin antigen by nitrocellulose dipstick tests (InBios International, Seattle, USA). The control subjects were free of any relevant infectious disease. Pregnant women, cases with other infections, healthy controls with a family history of VL and relatives of cases were excluded from the study. About 5.0 mls of full venous blood were collected from study subjects for serological and genetic studies. The samples were immediately transported to the lab and the serum samples were separated from whole blood and stored in the same type of tubes at -20°C until further use.

### DNA isolation and *FCN2* genotyping

Genomic DNA was extracted from peripheral blood leukocytes using the protocol described previously [27]. The reference genomic sequence was retrieved from the Ensembl database (www.ensembl.org). The five *FCN2* variants studied were PCR amplified from two genomic regions. The three promoter variants -986A>G, -602G>A and -4A>G were amplified by the primer pairs PromF-5'-ATTGAAGGAAAATCCGATGGG-3' and PromR-5'-GAAGCCACCAATCACGAAG-3', and the two exon-8 variants +6359 C>T and +6424 G>T were amplified using the primer pairs Exon8F-5'-CCAGCTCCCATGTCTAAAGG-3' and Exon8R-5'-TTACAAACCGTAGGGCCAAG-3'. Primers were designed by Primer-BLAST (http://www.ncbi.nlm.nih.gov/tools/primer-blast) and synthesized commercially (Eurofins, Bangalore, India). The target regions were amplified using an Emerald PCR master mix (TaKaRa, Shiga, Japan) and reactions were carried out in the ABI GeneAmp PCR system 9700. The thermal cycling parameters for both amplicons were: initial denaturation at 95°C for 5 minutes, followed by 35 cycles of denaturation at 94°C for 1 minute, annealing at 60°C for 30 seconds and elongation at 72°C for 1 minute. PCR products were purified using Exo-SAP-IT (USB-Affymetrix, Santa Clara, USA) and 1.0 μl of the products were directly used as templates for sequencing using the BigDye terminator (v.3.1) cycle sequencing kit (Applied Biosystems, Texas, USA) on an ABI 3730XL DNA Analyzer. Variations were identified by assembling DNA sequences with the reference sequence using AutoAssembler software (Applied Biosystems, Texas, USA) and were reconfirmed visually from their electropherograms.

### Ficolin-2 serological assay

Ficolin-2 levels were measured in the sera of VL cases (n = 166) and healthy controls (n = 85) using the human Ficolin-2 ELISA kit following manufacturer’s instructions (Hycult Biotech, Uden, The Netherlands). The detection limit of the assay was 16 ng/mL.

### Statistical analysis

Data were analyzed using the STATA software (Intercooled STATA, STATA Corp., College Station, TX, USA) and the level of significance was set to a *P* value of <0.05. Genotype or haplotype frequencies were calculated by simple gene counting and by expectation-maximum (EM) algorithm and the deviations from Hardy-Weinberg equilibrium were tested using the random-permutation procedure as implemented in the Arlequin v.3.5.1.2 software (http://lgb.unige.ch/arlequin). The linkage disequilibrium (LD) analysis was performed using Haploview v.3.2 (http://broadinstitute.org/haploview). Multivariate analysis was performed after adjustment with the confounding factors such as age, ethnicity and gender using the STATA software. In all comparisons, *P* values <0.05 were considered significant. Kruskal-Wallis or Wilcoxon-Mann-Whitney rank sum tests were applied wherever appropriate to analyze the correlation of serum ficolin-2 levels with *FCN2* variants and haplotypes by using the Kaleidagraph software (www.synergy.com).

## Results

### Association of *FCN2* variants with the risk of VL

**Fig 1:**
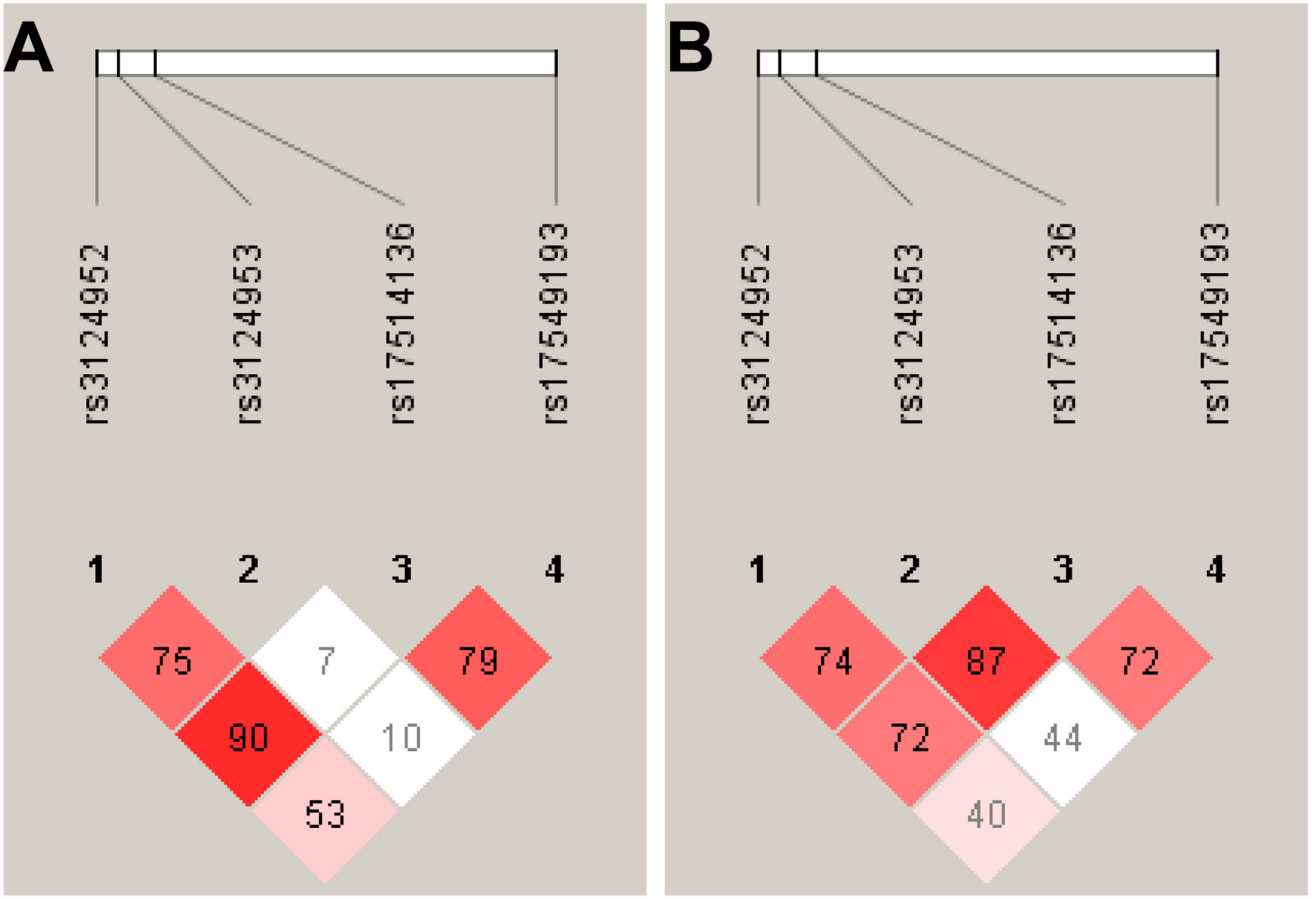
Linkage disequilibrium (LD) pattern of SNPs studied. (A): LD pattern of *FCN2* variants in visceral leishmaniasis cases and (B): LD pattern of *FCN2* variants in healthy controls. Numbers indicate the D’ value expressed as percentile. Open squares indicate the high degree of LD (LD coefficient D’ = 1) between pairs of variants. The red squares indicate pairs in strong LD with LOD scores.

The genotype and allele frequencies for the variants -986G>A, -602A>G, -4A>G and +6359C>T) in VL cases and controls were in Hardy-Weinberg equilibrium (*P*>0.05). This did not apply to the variant +6424G>T in VL cases. This variant was excluded from further analyses. The LD patterns of the *FCN2* variants are given in Fig 1. The LD plot indicates that the promoter variants -986G>A, -4A>G and the exon 8 variant +6359C>T were in strong LD with each other both in cases and controls. The variant -602A>G was in LD with +6359C>T only in controls. Significant differences were observed both in genotype and allele distributions between cases and controls for the non-synonymous variant +6359C>T (p.Thr236Met). The homozygous genotype *+6359TT* occurred more frequently among VL cases compared to controls after adjusting for age, sex and ethnicity (OR = 2.2, 95%CI = 1.23–7.25, *P* = 0.008), indicating that this variant was associated with an increased risk for *L*. *donovani* infection (Table 1). We observed a similar effect of the *+6359T* variant, when different genetic models are employed [Allelic: OR = 1.4, 95%CI = 1.02–1.94, *P* = 0.03; Recessive: OR = 2.2, 95%CI = 1.23–7.25, *P* = 0.008] (Table 1). The different genetic models indicate that the *+6359T* minor allele increases the susceptibility of *L*. *donovani* infection. The other investigated *FCN2* variants were not significantly associated with VL.

**Table 1:** Distribution of *FCN2* genotypes and alleles among visceral leishmaniasis cases and healthy controls.

rs17549193 (+6359C>T) (p.T236M)	VL Cases n = 204 (%)	Controls n = 223 (%)	OR (95% CI)	*P* ^#^ value
**Genotype**				
*CC*	110 (53.9)	134 (60.1)	1	Reference
*CT*	72 (35.2)	80 (35.8)	NA	NS
*TT*	22 (10.7)	9 (4.1)	2.2 (1.23–7.25)	0.008
**Allele**				
*C*	292 (71.5)	348 (78)	1	Reference
*T*	116 (28.5)	98 (22)	1.4 (1.02–1.94)	0.03
**Dominant**				
*CC*	110 (53.9)	134 (60.1)	1	Reference
*CT+TT*	94 (46.1)	89 (39.9)	NA	NS
**Recessive**				
*CC+CT*	182 (89.3)	214 (95.9)	1	Reference
*TT*	22 (10.7)	9 (4.1)	2.2 (1.23–7.25)	0.008

Note: CI, confidence interval; OR, odds ratio; NS, not significant; NA, not applicable. Percentage may not add up to 100 due to rounding errors

^#^ Adjusted *P* values for age, gender and ethnicity

The distribution of reconstructed *FCN2* haplotypes including variants -986G>A, -602A>G, -4A>G and +6359C>T are summarized in Table 2. Fifteen secretor haplotypes were observed. The four haplotypes FCN2*GGAC, *AGGT, *AAAC and *GGAT occurred at frequencies >10%. The reconstructed haplotype FCN2*AAAC was found more frequently in healthy controls compared to VL cases (OR = 0.59, 95%CI = 0.37–0.94, *P* = 0.023).

**Table 2:** Association of functional *FCN2* haplotypes and visceral leishmaniasis.

*FCN2* Haplotypes (-986/-602/ -4/+6359)	VL Cases n = 408(%)	Controls n = 446(%)	OR (95% CI)	*P* ^#^ value
*GGAC*	225 (55.1)	236 (52.9)	NA	NS
*AGGT*	54 (13.2)	53 (11.8)	NA	NS
***AAAC***	**36 (8.8)**	**62 (13.9)**	**0.59 (0.37–0.94)**	**0.023**
*GGAT*	24 (5.8)	20 (4.4)	NA	NS
*AAAT*	16 (3.9)	12 (2.6)	NA	NS
*AGAC*	14 (3.4)	24 (5.3)	NA	NS
*AGGC*	9 (2.2)	13 (2.9)	NA	NS
*AAGT*	8 (1.9)	0	NA	NA
*AGAT*	8 (1.9)	0	NA	NA
*GAAC*	7 (1.7)	10 (2.2)	NA	NS
*GGGT*	4 (0.9)	12 (2.6)	NA	NA
*GAAT*	2 (0.5)	0	NA	NA
*AAGC*	1 (0.2)	0	NA	NA
*GGGC*	0	3 (0.6)	NA	NA
*GAGT*	0	1 (0.2)	NA	NA

Note: CI, confidence interval; OR, odds ratio; NS, not significant; NA, not applicable. Percentage may not add up to 100 due to rounding errors

^#^Adjusted *P* values for age, gender and ethnicity

### Ficolin-2 serum levels and risk of VL

**Fig 2:**
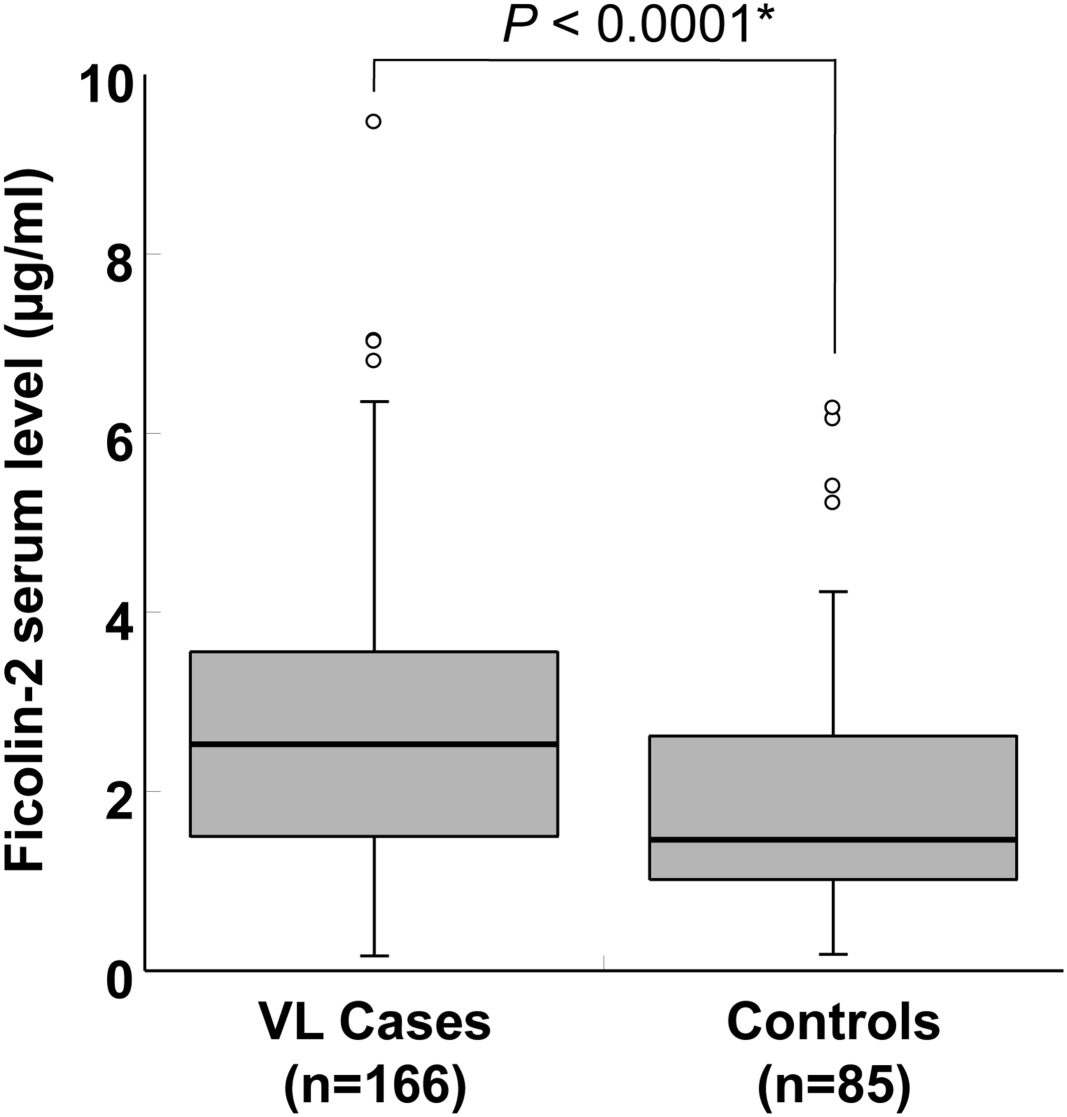
Distribution of ficolin-2 serum levels in visceral leishmaniasis cases and healthy controls. Box-plots illustrate medians with 25 and 75 percentiles with whiskers to 10 and 90 percentiles. *P values were calculated by multivariate analysis adjusted for age, gender and ethnicity. Numbers in parenthesis represent the number of samples.

**Fig 3:**
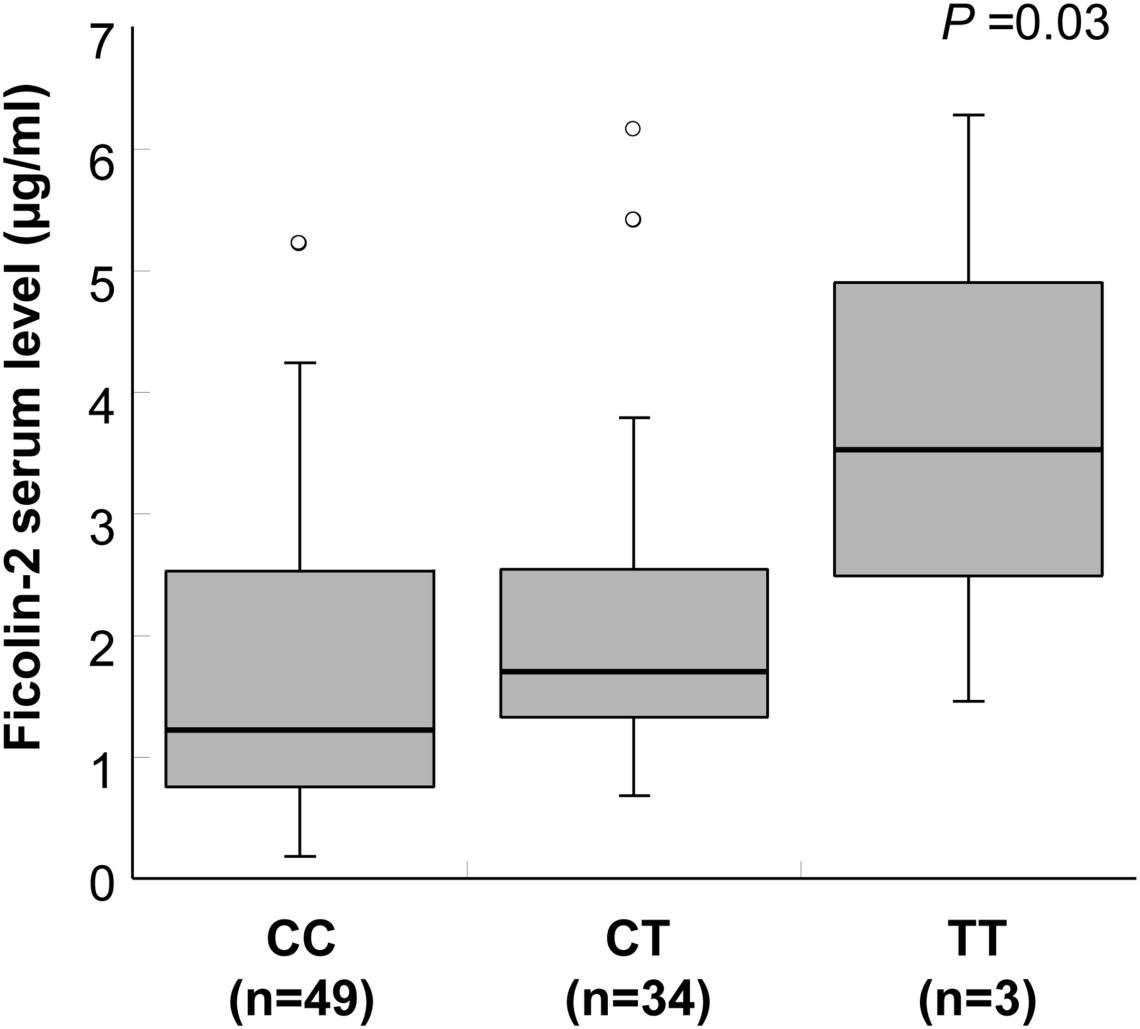
Distribution of ficolin-2 serum levels with +6359C>T variant in controls. Box-plots illustrate medians with 25 and 75 percentiles with whiskers to 10 and 90 percentiles. Ficolin-2 serum levels were measured and separated based on different genotypes of *FCN2* variant +6359C>T. *P =* 0.03 illustrated in the figure is calculated by Kruskal-Wallis rank sum test.

**Fig 4:**
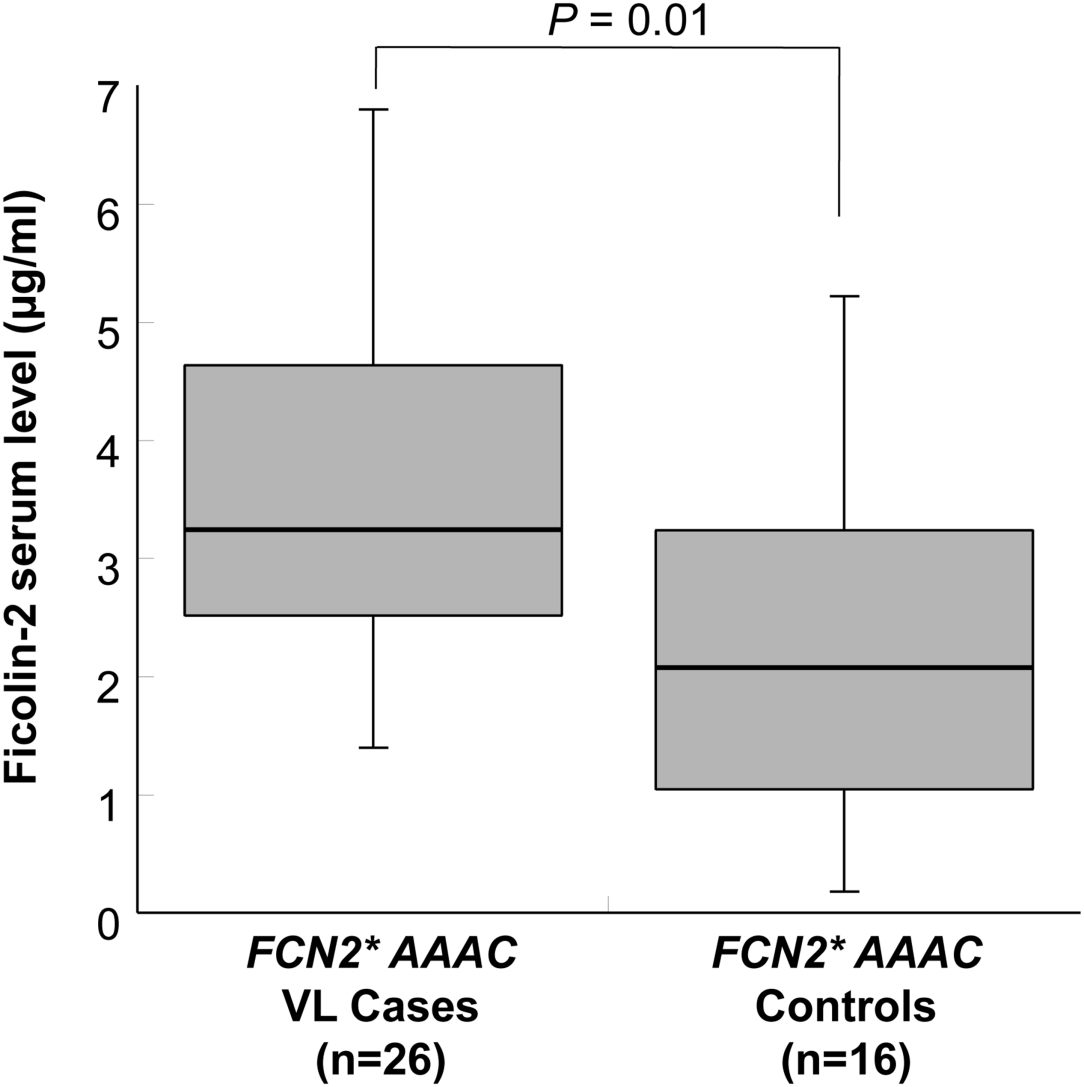
Distribution of ficolin-2 serum levels with FCN2*AAAC in VL cases and control. Box-plots illustrate medians with 25 and 75 percentiles with whiskers to 10 and 90 percentiles. *P =* 0.01 illustrated in the figure is calculated by Wilcoxon-Mann-Whitney rank sum test.

Ficolin-2 serum levels were significantly higher in VL cases (mean 2.77 μg/ml) compared to healthy controls (mean 1.94 μg/ml) (adjusted *P*<0.0001 for age, sex and ethnicity; Fig 2). Ficolin-2 levels are significantly distributed across different +6359 genotypes in controls (*P* = 0.03; Fig 3). Serum ficolin-2 levels in cases with the reconstructed FCN2*AAAC haplotypes were significantly higher than those measured in individuals of the control group (*P* = 0.01; Fig 4).

## Discussion

Visceral leishmaniasis develops when *L*. *donovani* parasites are successfully inoculated and survive the first-line attack of innate immune components such as phagocytes and the complement system. Indeed, these innate immune components play a major role both in the control and establishment of *L*. *donovani* infections [28]. Complement components including lectins are the primary molecules of the innate immune system to encounter inoculated metacyclic promastigotes. The early activation of the complement system during pathogen invasion occurs predominantly by the lectin pathway, as it is independent of a specific antibody response. Moreover, it prompts the activation of the alternative pathway [29]. The lectin pathway protein MBL induces opsonophagocytosis by depositing C3b on the surface of *Leishmania* which is crucial for parasite survival and multiplication [13,14]. We assume that, as ficolins are functionally similar to MBL, they equally influence the outcome of VL. No study so far, however, has focused on the role of ficolin-2 in VL. We studied the contribution of ficolin-2 serum levels and of *FCN2* functional variants in VL.

The structural variant +6359C>T (p.T236M) in the fibrinogen-like domain of the *FCN2* gene confers relative susceptibility to VL. The finding remains consistent in recessive and allelic genetic models. The computational prediction revealed that the T236M substitution has a major impact on the physiochemical property of ficolin-2 [30]. In addition, the *+6359T* allele was found associated with higher ficolin-2 serum levels [31] and the observation was reconfirmed in a cohort of neonates [32]. We also observed a similar effect of the *+6359T* allele in controls, but not in cases. Our results inferred that ficolin-2 serum levels were modulated significantly by the infection in VL cases rather than by *FCN2* variants. Moreover, the ficolin-2 protein with T236M substitution had a markedly decreased binding capacity to acetylated agarose beads. Therefore, this structural variant is believed to alter the binding properties of the protein to recognize invading pathogens [24,33,34]. These reports indicate that individuals with higher ficolin-2 serum levels and altered binding capacities might favor *L*. *donovani* invasion into macrophages and the development of VL. Previous studies have also reported that +6359C>T in the *FCN2* gene is a risk factor for staphylococcal peritonitis in continuous ambulatory peritoneal dialysis cases [35] and for bloodstream infections in kidney transplant recipients [36].

In *FCN2* gene-association studies, haplotype analyses should be taken into account as they may influence disease susceptibility [37]. Our *FCN2* haplotypes revealed that the FCN2*AAAC haplotype frequency was higher among controls than in VL cases, indicating that individuals with this haplotype had a diminished probability to develop VL. The FCN2*AAAC haplotype harbors the *+6359C* major allele, which accounts for reduced ficolin-2 levels [31,32]. In light of these observations, it is evident that the *FCN2* genetic factors that contribute to low ficolin-2 level decrease the risk of VL. *FCN2* promoter haplotypes did not show any differences among groups, suggesting the relative contribution of the +6359C>T genotype in Indian VL.

Ficolin-2 serum levels were elevated in VL cases compared to controls, indicating that ficolin-2 is a susceptibility factor. The result is in accordance with a study published previously [15]. Corresponding results were also observed in infections with *Mycobacterium* spp., where higher MBL serum levels increased the risk of infection [38–40]. The proposed mechanism may be that intracellular parasites abuse C3 opsonization and enhance opsonaophagocytosis by monocytes/macrophages to avoid complement attacks. Any increase in the MBL and ficolin levels in turn may enhance complement activation and, thus, the probability of parasitization by depositing C3b on parasite surfaces [41]. Our observation supports this notion as cases with VL had higher ficolin-2 levels than uninfected controls. Nevertheless, discordant results were reported for ficolin-2 in tuberculosis and Chagas disease, where cases presented lower ficolin-2 plasma levels than did controls [22,42]. No clear mechanism is proposed to address the conflictive observations of functionally similar proteins in intracellular habitant infections. In addition, the recognition and interaction of mannose binding lectin (MBL) with *Leishmania* parasites are well established [13,14] and ficolins were shown to be functional analogous to MBL [16]. However, a limitation of our study is that there is a lack of data showing the interaction of ficolin-2 with *L*. *donovani*. Nevertheless, our earlier study demonstrated the genetic association of *FCN2* polymorphism with cutaneous leishmaniasis in Syrian population [17].

In conclusion, our results show that the *FCN2* +6359C>T variant is associated with increased susceptibility to VL and that the FCN2*AAAC haplotype is associated with relative protection. Higher serum ficolin-2 levels were observed in cases with VL than among controls.

## References

[1] Ready PD (2014) Epidemiology of visceral leishmaniasis. Clin Epidemiol 6: 147–154. 10.2147/CLEP.S44267 [doi];clep-6-147 [pii]. 24833919 PMC4014360

[2] Mathers CD, Ezzati M, Lopez AD (2007) Measuring the burden of neglected tropical diseases: the global burden of disease framework. PLoS neglected tropical diseases 1: e114 10.1371/journal.pntd.0000114 18060077 PMC2100367

[3] Chappuis F, Sundar S, Hailu A, Ghalib H, Rijal S, Peeling RW, et al (2007) Visceral leishmaniasis: what are the needs for diagnosis, treatment and control? Nature reviews Microbiology 5: 873–882. 10.1038/nrmicro1748 17938629

[4] Desjeux P (1996) Leishmaniasis. Public health aspects and control. Clinics in dermatology 14: 417–423. 8889319 10.1016/0738-081x(96)00057-0

[5] Hotez PJ, Remme JH, Buss P, Alleyne G, Morel C, Breman JG (2004) Combating tropical infectious diseases: report of the Disease Control Priorities in Developing Countries Project. Clinical infectious diseases: an official publication of the Infectious Diseases Society of America 38: 871–878. 10.1086/382077 14999633

[6] Singh SP, Reddy DC, Rai M, Sundar S (2006) Serious underreporting of visceral leishmaniasis through passive case reporting in Bihar, India. Tropical medicine & international health: TM & IH 11: 899–905. 10.1111/j.1365-3156.2006.01647.x 16772012

[7] Muniaraj M (2014) The lost hope of elimination of Kala-azar (visceral leishmaniasis) by 2010 and cyclic occurrence of its outbreak in India, blame falls on vector control practices or co-infection with human immunodeficiency virus or therapeutic modalities? Tropical parasitology 4: 10–19. 10.4103/2229-5070.129143 24754021 PMC3992795

[8] Sacks D, Kamhawi S (2001) Molecular aspects of parasite-vector and vector-host interactions in leishmaniasis. Annual review of microbiology 55: 453–483. 10.1146/annurev.micro.55.1.453 11544364

[9] Moradin N, Descoteaux A (2012) Leishmania promastigotes: building a safe niche within macrophages. Frontiers in cellular and infection microbiology 2: 121 10.3389/fcimb.2012.00121 23050244 PMC3445913

[10] Descoteaux A, Turco SJ (1999) Glycoconjugates in Leishmania infectivity. Biochimica et biophysica acta 1455: 341–352. 10571023 10.1016/s0925-4439(99)00065-4

[11] Flandin JF, Chano F, Descoteaux A (2006) RNA interference reveals a role for TLR2 and TLR3 in the recognition of Leishmania donovani promastigotes by interferon-gamma-primed macrophages. European journal of immunology 36: 411–420. 10.1002/eji.200535079 16369915

[12] Descoteaux A, Turco SJ (2002) Functional aspects of the Leishmania donovani lipophosphoglycan during macrophage infection. Microbes and infection / Institut Pasteur 4: 975–981. 12106791 10.1016/s1286-4579(02)01624-6

[13] Ambrosio AR, De Messias-Reason IJ (2005) Leishmania (Viannia) braziliensis: interaction of mannose-binding lectin with surface glycoconjugates and complement activation. An antibody-independent defence mechanism. Parasite immunology 27: 333–340. 10.1111/j.1365-3024.2005.00782.x 16149991

[14] Green PJ, Feizi T, Stoll MS, Thiel S, Prescott A, McConville MJ (1994) Recognition of the major cell surface glycoconjugates of Leishmania parasites by the human serum mannan-binding protein. Molecular and biochemical parasitology 66: 319–328. 7808481 10.1016/0166-6851(94)90158-9

[15] Santos IK, Costa CH, Krieger H, Feitosa MF, Zurakowski D, Fardin B et al (2001) Mannan-binding lectin enhances susceptibility to visceral leishmaniasis. Infection and immunity 69: 5212–5215. 10.1128/IAI.69.8.5212-5215.2001 11447210 PMC98624

[16] Ren Y, Ding Q, Zhang X (2014) Ficolins and infectious diseases. Virologica Sinica 29: 25–32. 10.1007/s12250-014-3421-2 24452543 PMC8206374

[17] Assaf A, Hoang TV, Faik I, Aebischer T, Kremsner PG, Kun JF et al (2012) Genetic evidence of functional ficolin-2 haplotype as susceptibility factor in cutaneous leishmaniasis. PloS one 7: e34113 10.1371/journal.pone.0034113 22457818 PMC3311577

[18] Matsushita M (2010) Ficolins: complement-activating lectins involved in innate immunity. Journal of innate immunity 2: 24–32. 10.1159/000228160 20375620

[19] Matsushita M (2013) Ficolins in complement activation. Molecular immunology 55: 22–26. 10.1016/j.molimm.2012.08.017 22959617

[20] Hoang TV, Toan NL, Song lH, Ouf EA, Bock CT, Kremsner PG et al (2011) Ficolin-2 levels and FCN2 haplotypes influence hepatitis B infection outcome in Vietnamese patients. PLoS One 6: e28113 10.1371/journal.pone.0028113 [doi];PONE-D-11-14849 [pii]. 22140517 PMC3222672

[21] Ouf EA, Ojurongbe O, Akindele AA, Sina-Agbaje OR, Van TH, Adeyeba AO et al (2012) Ficolin-2 levels and FCN2 genetic polymorphisms as a susceptibility factor in schistosomiasis. J Infect Dis 206: 562–570. jis396 [pii]; 10.1093/infdis/jis396 [doi]. 22693230

[22] Luz PR, Boldt AB, Grisbach C, Kun JF, Velavan TP, Messias-Reason IJ (2013) Association of L-ficolin levels and FCN2 genotypes with chronic Chagas disease. PloS one 8: e60237 10.1371/journal.pone.0060237 23593180 PMC3617223

[23] Endo Y, Matsushita M, Fujita T (2011) The role of ficolins in the lectin pathway of innate immunity. The international journal of biochemistry & cell biology 43: 705–712. 10.1016/j.biocel.2011.02.003 21315829

[24] Hummelshoj T, Munthe-Fog L, Madsen HO, Fujita T, Matsushita M, Garred P (2005) Polymorphisms in the FCN2 gene determine serum variation and function of Ficolin-2. Human molecular genetics 14: 1651–1658. 10.1093/hmg/ddi173 15879437

[25] Munthe-Fog L, Hummelshoj T, Hansen BE, Koch C, Madsen HO, Skjodt K et al (2007) The impact of FCN2 polymorphisms and haplotypes on the Ficolin-2 serum levels. Scandinavian journal of immunology 65: 383–392. 10.1111/j.1365-3083.2007.01915.x 17386030

[26] Krarup A, Thiel S, Hansen A, Fujita T, Jensenius JC (2004) L-ficolin is a pattern recognition molecule specific for acetyl groups. The Journal of biological chemistry 279: 47513–47519. 10.1074/jbc.M407161200 15331601

[27] Thangaraj K, Joshi MB, Reddy AG, Gupta NJ, Chakravarty B, Singh L (2002) CAG repeat expansion in the androgen receptor gene is not associated with male infertility in Indian populations. J Androl 23: 815–818. 12399527

[28] Laurenti MD, Corbett CE, Sotto MN, Sinhorini IL, Goto H (1996) The role of complement in the acute inflammatory process in the skin and in host-parasite interaction in hamsters inoculated with Leishmania (Leishmania) chagasi. International journal of experimental pathology 77: 15–24. 8664142 10.1046/j.1365-2613.1996.958096.x PMC2691615

[29] Cestari I, Evans-Osses I, Schlapbach LJ, de Messias-Reason I, Ramirez MI (2013) Mechanisms of complement lectin pathway activation and resistance by trypanosomatid parasites. Molecular immunology 53: 328–334. 10.1016/j.molimm.2012.08.015 23063472

[30] Hummelshoj T, Munthe-Fog L, Madsen HO, Garred P (2008) Functional SNPs in the human ficolin (FCN) genes reveal distinct geographical patterns. Molecular immunology 45: 2508–2520. 10.1016/j.molimm.2008.01.003 18289682

[31] Cedzynski M, Nuytinck L, Atkinson AP, St Swierzko A, Zeman K, Szemraj J et al (2007) Extremes of L-ficolin concentration in children with recurrent infections are associated with single nucleotide polymorphisms in the FCN2 gene. Clinical and experimental immunology 150: 99–104. 10.1111/j.1365-2249.2007.03471.x 17680820 PMC2219292

[32] Kilpatrick DC, St Swierzko A, Matsushita M, Domzalska-Popadiuk I, Borkowska-Klos M, Szczapa J et al (2013) The relationship between FCN2 genotypes and serum ficolin-2 (L-ficolin) protein concentrations from a large cohort of neonates. Human immunology 74: 867–871. 10.1016/j.humimm.2013.04.011 23619474

[33] Herpers BL, Immink MM, de Jong BA, van Velzen-Blad H, de Jongh BM, van Hannen EJ (2006) Coding and non-coding polymorphisms in the lectin pathway activator L-ficolin gene in 188 Dutch blood bank donors. Molecular immunology 43: 851–855. 10.1016/j.molimm.2005.06.035 16076493

[34] Ma YJ, Doni A, Hummelshoj T, Honore C, Bastone A, Mantovani A et al (2009) Synergy between ficolin-2 and pentraxin 3 boosts innate immune recognition and complement deposition. The Journal of biological chemistry 284: 28263–28275. 10.1074/jbc.M109.009225 19632990 PMC2788878

[35] Meijvis SC, Herpers BL, Endeman H, de Jong B, van Hannen E, van Velzen-Blad H et al (2011) Mannose-binding lectin (MBL2) and ficolin-2 (FCN2) polymorphisms in patients on peritoneal dialysis with staphylococcal peritonitis. Nephrology, dialysis, transplantation: official publication of the European Dialysis and Transplant Association—European Renal Association 26: 1042–1045. 10.1093/ndt/gfq474 20682603

[36] Wan QQ, Ye QF, Zhou JD (2013) Mannose-binding lectin 2 and ficolin-2 gene polymorphisms influence the susceptibility to bloodstream infections in kidney transplant recipients. Transplantation proceedings 45: 3289–3292. 10.1016/j.transproceed.2013.05.008 24182802

[37] de Messias-Reason I, Kremsner PG, Kun JF (2009) Functional haplotypes that produce normal ficolin-2 levels protect against clinical leprosy. The Journal of infectious diseases 199: 801–804. 19434912 10.1086/597070

[38] De Messias-Reason IJ, Boldt AB, Moraes Braga AC, Von Rosen Seeling Stahlke E, Dornelles L, Pereira-Ferrari L et al (2007) The association between mannan-binding lectin gene polymorphism and clinical leprosy: new insight into an old paradigm. The Journal of infectious diseases 196: 1379–1385. 10.1086/521627 17922403

[39] Hoal-Van Helden EG, Epstein J, Victor TC, Hon D, Lewis LA, Beyers N et al (1999) Mannose-binding protein B allele confers protection against tuberculous meningitis. Pediatric research 45: 459–464. 10.1203/00006450-199904010-00002 10203135

[40] Soborg C, Madsen HO, Andersen AB, Lillebaek T, Kok-Jensen A, Garred P (2003) Mannose-binding lectin polymorphisms in clinical tuberculosis. The Journal of infectious diseases 188: 777–782. 10.1086/377183 12934195

[41] Dommett RM, Klein N, Turner MW (2006) Mannose-binding lectin in innate immunity: past, present and future. Tissue Antigens 68: 193–209. TAN649 [pii]; 10.1111/j.1399-0039.2006.00649.x [doi]. 16948640 PMC7169806

[42] Luo F, Sun X, Wang Y, Wang Q, Wu Y, Pan Q et al (2013) Ficolin-2 defends against virulent Mycobacteria tuberculosis infection in vivo, and its insufficiency is associated with infection in humans. PloS one 8: e73859 10.1371/journal.pone.0073859 24040095 PMC3767610

